# The Washington Group Short Set of Functioning as a Global Disability Data Collection Tool: A Discussion on Its Comparability With a Local Disability Index From Japan

**DOI:** 10.7759/cureus.102282

**Published:** 2026-01-25

**Authors:** Takashi Saito, Kumiko Imahashi

**Affiliations:** 1 Department of Social Rehabilitation, Research Institute of National Rehabilitation Center for Persons With Disabilities, Tokorozawa, JPN

**Keywords:** disability, disability certificate, disability statistics, functional assessment, intractable disease, japan, washington group short set questionnaire on functioning

## Abstract

Disability is a global public health issue. To tackle the issue, internationally comparable disability data collection tools are needed. Against the backdrop of the global demand, the Washington Group Short Set on Functioning (WG-SS) has been developed and adapted globally. It is reasonable, from a global perspective, to use the internationally standardized tool to capture the current situation across the world. However, use of the tool may not necessarily be reasonable from a local perspective. In Japan, traditionally, persons with a legal disability certificate or an intractable disease are recognized as persons with disabilities. Previous studies based on secondary analyses of a national representative data set from Japan, however, suggested that a significant portion of them were not captured by the WG-SS; the percentages of persons being captured by the WG-SS among persons with a legal disability certificate and intractable disease were approximately 45% and 40%, respectively. Disability statistics based on the WG-SS may not fully reflect the whole picture of individuals with a legal disability certificate and intractable diseases in Japan. Therefore, utilization of the disability data for policy- making on Japanese local disability issues would need to be done with careful considerations over the limitations. These kinds of considerations may also be needed among not only Japanese stakeholders but also those in other countries where similar potential disparities exist between the WG-SS and the local disability index. A better understanding of the comparability between the WG-SS and the local disability index is essential for effective utilization of the WG-SS from a local perspective.

## Editorial

Disability is a global public health issue. Disability potentially results in a lower living standard and poverty among people through disability-induced limitations for social participation, which consequently leads to social inequity between people with and without disabilities. To tackle the global issue, internationally comparable and high-quality disability data collection tools that can lay the groundwork for envisaging the current situation and developing solutions are needed. The Washington Group Short Set of Functioning (WG-SS), an internationally comparable disability data collection tool, was developed and adapted globally since 2006 against the backdrop [1].

The primary purpose of the WG-SS is to estimate disability prevalence and to present disaggregating key indicators of social participation between people with and without disabilities. The WG-SS consists of six questions on universal basic functions: seeing, hearing, walking, self-care, cognition, and communication. The questions have a set of uniform response categories: no difficulty, some difficulty, a lot of difficulty, and cannot do it at all. Respondents who answer ‘a lot of difficulty’ or ‘cannot do it at all’ to at least one of the six questions are considered persons who are at greater risk for limitations in participation, i.e., people with disabilities. This cut-off, or threshold, is recommended for international comparison by the developers of the WG-SS.

More than 20 countries have published reports, titled “Country Disability Reports,” on disability prevalence and disaggregating key indicators using the WG-SS [2]. The reports aim to disseminate basic, comparable disability statistics globally. For instance, according to the Country Disability Report of the U.S., the age-adjusted percentage of persons with disabilities is 8.3% in the U.S. [2]. Regarding educational attainment as “less than a high school education,” a significant gap was reported: adults with disabilities (15.2%) and without disabilities (9.5%) [2]. The information provided by the reports can be a reference for global communities when reviewing disability policy in each country and region.

We understand that it is reasonable from a global perspective to use an internationally standardized tool to capture the current situation across the world. However, we would like to argue that the use of the internationally standardized tool may not necessarily be reasonable from a local perspective. Each country has its own unique index for defining persons with disabilities and estimating disability prevalence. The disability prevalence, consequently, is used to develop and deliver social welfare programs for persons with disabilities in each country. However, there may be a significant disparity between disability statistics based on an internationally standardized tool and a local index. In that case, caution is needed when interpreting the disability statistics based on an internationally standardized tool, like the WG-SS.

Japan has its own unique index for defining persons with disabilities. Traditionally, persons with a legal disability certificate or an intractable disease are recognized as persons with disabilities in Japan. The legal disability certificate systems are categorized into three types: physical disability certificate, medical rehabilitation handbook (intellectual disability certificate), and mental disability certificate (Table 1) [3,4]. These certificates are issued for persons with disabilities who meet the severity criteria stipulated by relevant laws or notifications. Legal disability certificate holders are entitled to receive designated medical services, welfare services, or financial support. As of 2023, the number of physical disability certificate holders, medical rehabilitation handbook holders, and mental disability certificate holders is 4,783,069, 1,281,469, and 1,438,093, respectively [3]. Moreover, the Japanese government designates more than 300 intractable diseases as “designated intractable diseases.” Persons who are diagnosed with any of the designated intractable diseases by a designated medical doctor are issued a medical care recipient certificate (Table 1). The certificate entitles holders to receive relevant medical services with partial out-of-pocket expenditure. As of 2024, the number of medical care recipient certificate holders is 1,121,462.

**Table 1:** System of the legal disability certificates and designated intractable diseases in Japan NOTE: This table was independently created by the authors. ^†^ Source of information is the Ministry of Health, Labour and Welfare of Japan [3]. ^††^ Source of information is the Japan Intractable Disease Information Center [4].

	Disability certificate ^†^			Designated intractable diseases ^††^
	Physical disability certificate	Medical rehabilitation handbook (intellectual disability certificate)	Mental disability certificate	Medical care recipient certificate
Age	All Ages	All Ages	All Ages	All Ages
Number of certificate holders	4,783,069 as of 2023	1,281,469 as of 2023	1,438,093 as of 2023	1,121,462 as of 2024
Legal basis	Act for the Welfare of Persons with Physical Disabilities (1949)	There is no legal definition of intellectual disabilities. The certificate system is in accordance with the notification issued by the Administrative Vice-minister in September 1973: *Regarding Certificate System for Persons with Intellectual Disabilities*.	Act on Mental Health and Welfare for Persons with Mental Disorders or Disabilities (1950)	Act on Medical Care for Patients with Intractable Diseases (2014)
Medical diagnosis	Required	Not required	Required	Required
Types of disabilities	Visual impairment: visual impairment. Hearing and equilibrium impairment: hearing impairment, equilibrium impairment. Speech impairment: voice, speech, and language disorders. Mobility impairment: upper limb impairment, lower limb impairment, trunk impairment, upper-limb impairment induced by brain function disorder, mobility impairment induced by brain function disorder. Visceral impairment: cardiac disorder, renal disorder, respiratory disorder, bladder or rectal disorder, small intestinal disorder, immunological disorder by human immunodeficiency virus, hepatic disorder.	Intellectual disabilities that meet the severity criteria based on the relevant notifications.	Mental disabilities that meet the severity criteria stipulated by the relevant law and notifications. Examples: schizophrenia, epilepsy, mood disorders, higher brain dysfunction, substance use disorder, developmental disabilities, Stress-related disabilities.	As of 2025, 348 intractable diseases are specified by the Japanese government as designated intractable diseases. Characteristics of the diseases are: having no known treatment and of unknown causes, rare disease (prevalence is approximately 0.1% in Japan), requiring long-term care, and having established certain standards for diagnosis by objective indicators.
Degree of disabilities	Grade 1 (severe) to Grade 7 (mild). Each type of disability has unique severity criteria stipulated by the relevant law.	Severe (Grade A): Persons with an IQ below approximately 35 who have either of the intellectual disability: a) Requiring assistance or supervision for daily living activities and experiencing significant difficulty in adapting to social life. b ) Having frequent problematic behaviors (example: abnormal eating habits or excitation). Other (Grade B): Others who are not eligible for the criteria in Grade A. Note: Differences in the grade of intellectual disability exist among prefectures.	Grade 1: Individuals with a disorder that prevents them from conducting daily activities. Grade 2: Individuals with a disorder that severely limits their daily activities. Grade 3: Individuals with a disorder that limits their daily or social activities.	Depending on the diseases.
Benefits	Examples: independent living medical care, assistive medical devices, tax deduction	Examples: Child support allowance, tax deduction.	Examples: Independent living medical care, tax deduction.	Payment of specific medical expenses.

If a significant portion of persons with a legal disability certificate or intractable disease are not captured by the WG-SS, disability statistics based on the internationally standardized tool should be interpreted with caution from a local Japanese perspective. According to a study that is based on a secondary analysis set of Japanese representative samples of people with disabilities, percentages of persons being captured by the WG-SS among persons with a legal disability certificate and intractable disease were approximately 45% and 40%, respectively [5]. Moreover, it is suggested that legal disability certificate holders with mild impairments were less likely to be captured by the WG-SS than those with severe impairments; namely, disability statistics based on the WG-SS may systematically exclude legal disability certificate holders with mild impairments in Japan [6]. These previous studies suggested that disability statistics based on the WG-SS were not comparable with those based on the Japanese local disability index. Namely, Japanese disability statistics based on the WG-SS may not fully reflect the whole picture of individuals with a legal disability certificate and intractable diseases in Japan. The disparity may be attributed to differences in the way of defining disability; a legal disability certificate or intractable disease is based on a medical diagnosis made by a medical doctor, and WG-SS is based on the self-reporting subjective difficulty of individuals.

To the best of our knowledge, Country Disability Reports of Japan have not yet been published at the time of writing this paper. In the near future, however, the report may be published and available globally. When interpreting the disability data in the report, Japanese local stakeholders need to understand the disability data, taking into account inherent characteristics. For instance, the disability data would be a good reference to capture the current Japanese situation regarding issues of people with disabilities from a global perspective. However, the Japanese stakeholders need to understand that the disability data might underestimate the disability prevalence and disability-based inequity in Japan. Therefore, utilization of the disability data for policy-making on Japanese local disability issues would need to be done with careful consideration of the limitations. These kinds of considerations may also need to be considered by not only Japanese stakeholders but also by those in other countries where similar potential disparities exist between the WG-SS and the local disability index.

Again, from a global perspective, the internationally comparable disability data collection tool is reasonable and essential to capture the current situation and to develop solutions globally. We do not intend to object to the idea. When interpreting the disability statistics from a local perspective, however, some reservations must be made for possible disparity between the disability statistics based on the WG-SS and the local disability index. A better understanding of the comparability is essential for more effective utilization of the WG-SS.
